# Students' Perceptions on Online Clinical Learning amid the COVID-19 Pandemic in an Institution of Higher Learning: A Qualitative Inquiry

**DOI:** 10.1155/2023/4901661

**Published:** 2023-07-31

**Authors:** Jean Mukasa, Doreen Macherera Mukona, Smitha Joseph, Anupama Kanissery, Joemol James, Maribeth Carpio Tabay, Salimbabu Abdulla, Hussam Al Amoor

**Affiliations:** ^1^Fatima College of Health Sciences, Department of Nursing, Ajman, UAE; ^2^Fatima College of Health Sciences, General Requirements Department, Ajman, UAE

## Abstract

Institutions of learning have been disrupted globally with serious implications for clinical teaching for students of health professions. The purpose of our study was to explore the perceptions of students towards online clinical teaching during the COVID-19 pandemic at Fatima College of Health Sciences. This was a descriptive qualitative study conducted on a purposive sample of 25 students from 24 June to 30 August 2020. The sample size was determined by data saturation. These were mainly nursing students in their 2nd to the 4th years of study. Students are required to have experiences, of stipulated nature and duration, in various specialty clinical settings throughout the clinical years of their programs. Approval for the study was granted by the Fatima College Research Ethics Committee (approval number: INTSTF010BSN20). The research was conducted according to the requirements of the Declaration of Helsinki. Data were collected through online semistructured questionnaires. Prospective participants were sent a soft copy of the informed consent document, and consent was indicated by clicking an “agree” link on the page that took them to the questionnaire. All participants were informed of their freedom to either participate in the study or not, without any penalty and were assured of their confidentiality. The questionnaires were kept in a password-protected file to which the researchers had sole access. Manual thematic analysis was done following the stages of organisation, familiarisation, transcription, coding, developing a thematic framework, indexing, displaying, and reporting. The major themes identified were the unfamiliar experience, challenges of online clinical learning, and possible solutions. Challenges of online clinical learning are multifaceted and require concerted multidisciplinary efforts to resolve. Nursing institutions, ours included, must develop flexible education systems that will be able to thrive in crisis and other unforeseeable circumstances.

## 1. Introduction

The recent outbreak of the novel coronavirus pandemic in China has become a serious threat internationally, causing the closure of many organisations and institutions. Governments worldwide have instituted several steps to control the spread of disease, including the closure of educational institutions as part of national lockdowns imposed on many countries. The closure of schools resulted in the transfer of teaching and learning from the classroom to the home-based online mode. This, unfortunately, has had an impact on education and mental health status of students and academic staff globally [1]. The sudden shift in methods of instruction from face-to-face instruction to online has created a host of challenges for both instructors and students alike [2, 3]. In spite of the online mode of delivering classes not being a new technology in education, it demanded faculty members to rapidly transit from the traditional methods conventionally used in institutions of learning [4].

Healthcare systems have been disrupted globally with serious implications for clinical teaching for students of health professions. Some institutions have completely suspended clinical placements with the hope of mitigating viral transmission [5]. Health workers who are also involved in clinical teaching could not be available for meaningful clinical learning [3, 6], consequently creating major challenges in the development of high-quality methods of education, assessment, and evaluation. Health workers were among the hardest hit by the pandemic [7, 8]. In addition, this compromised the quality of clinical teaching, as students could not achieve their competences within the usual timeframes [9]. The clinical environment is the best place for teaching psychomotor skills in most health professions' education such as nursing. Though some skills can be taught in a nonclinical setting, through simulation, bedside training in nursing remains the cornerstone of nursing education.

Institutions of higher learning in United Arab Emirates (UAE), including the Fatima College of Health Sciences, experienced similar shifts where there was an urgent need to shift to a wholly online mode of teaching. The college offers health professions programs, namely, Nursing, Physiotherapy, Pharmacy, Emergency Health, and Radiological studies. These are 4-year programs divided into 2, the first preclinical year and 3 subsequent clinical years. During the clinical years, the students are placed in various areas, in healthcare institutions, for the required clinical experiences. Due to the lockdowns instituted worldwide, students could not be placed in clinical settings or attend laboratory sessions on campus. Because of the need to maintain continuity of health professions education, online clinical teaching, though relatively new, was the only feasible method of instruction. This mode of instruction involves using computer technology to deliver training, including technology-supported learning either online, offline, or both [10]. Before the pandemic, online clinical teaching was not conventionally preferred as a method of instruction for the development of psychomotor skills [10].

Many modifications were required in the assessment criteria to fit the online mode of instruction. The abruptness of the transition, coupled with the relative lack of experience for both instructors and students, posed a major challenge for teaching, learning, and assessment. Some documented challenges include the high probability of academic dishonesty, compromised reliability of assessment tools due to the lack of close monitoring [11, 12], and failure to effectively cater for laboratory assessments, clinical evaluation, and other clinical performance tests that are central to medical, paramedical, nursing, and other clinical health science courses.

While a number of studies have elaborated on the challenges experienced from transitioning from the traditional to online pedagogical approaches, there is a need to comprehensively appraise transition regarding clinical teaching. There are no universally accepted or applicable guidelines for transitioning clinical teaching, and it is very important to do so without compromising the quality of education. It is hoped that findings from this paper will contribute to the development of guidelines for transitioning to online clinical teaching, amid the ongoing COVID-19 pandemic. The purpose of this study was, therefore, to explore nursing students' perceptions on online clinical learning amid the COVID-19 pandemic at a tertiary institution of health sciences in the United Arab Emirates.

## 2. Materials and Methods

This was a descriptive qualitative study conducted at a college of health sciences. The study sought to explore the perceptions of students on online clinical teaching during the COVID-19 pandemic. Purposive sampling was performed. Data collection commenced on the 24th of June 2020 and proceeded until the 30th of August 2020 when data saturation was achieved, at 25 students. The whole target population comprised about 180 students. The participants were mainly nursing students in their 2nd to the 4th years of study, which are the clinical years according to the various programs offered at the college. Students are required to have experiences, of stipulated nature and duration, in various clinical settings throughout the programs. Examples are Maternal and Child Health, Critical Care, Community, and Mental and Medical Surgical Nursing. Approval for the study was granted by the Fatima College Research Ethics Committee (approval number: INTSTF010BSN20). The research was conducted according to the requirements of the Declaration of Helsinki [13]. Data were collected using an online semistructured questionnaire because all teaching and learning occurred online owing to COVID-19 movement restrictions. Prospective participants were sent a soft copy of the informed consent document, and consent was indicated by clicking an “agree” link on the page that took them to the questionnaire. Data saturation was reached at 25. The participants were informed that they could decline to participate or withdraw from the study at any time without any penalty. They were assured of their confidentiality, and no identifying information was included on the completed questionnaires. The questionnaires were kept in a password-protected file to which the researchers had sole access. Each online questionnaire lasted about 30 minutes to complete. Manual thematic analysis was performed following the stages of organisation, familiarisation, transcription, coding, developing a thematic framework, indexing, displaying, and reporting as recommended by Braun and Clarke [14, 15]. Minor and major themes were identified, and data were presented in the form of themes. Trustworthiness was ensured by observing credibility (member checks, prolonged engagement, thick description of events, and triangulation), dependability, confirmability, and transferability. The researchers repeatedly sort clarity from the participants regarding the codes, subthemes, and main themes throughout the process. The whole research process is well explained for transferability and confirmability.

## 3. Results

This section presents the findings of the study. This was a descriptive qualitative analysis of 25 nursing students at a tertiary institution.

### 3.1. Demographic Data

Table 1 presents the demographic characteristics of the respondents. All respondents were from Ajman Campus, were female, and were all Native Emirati students. Majority of students (88%) were aged 17–24 years.

Table 2 presents themes, as they emerged from codes and the respective subthemes. Three main themes were identified from the data analysis, namely, the unfamiliar experience, challenges of online clinical learning, and possible solutions.

### 3.2. The Unfamiliar Experience

Many respondents cited that the experience of online clinical learning was unfamiliar and not stimulating. The experience was largely unfamiliar, and the lack of social interaction and moral support made it unpleasant. The following excerpts encapsulate the experiences verbatim.

For example, one student displayed a lot of frustration on multiple issues relating to the administration, technical issues, and so on as cited

*I have noticed also that our grades are going down not as usual time in the collage. The technical issue is another story our time is wasting in the exam because of these issues and when we discussed with our instructors, no one understands us. I am really want to complete the remaining courses for the next academic year in the collage I need to practice on the labs and go again to the clinical. A lot of stress during online classes hundreds of quizzes at the same time and again and again when we discuss these issues no one care, they told us that it's not the first time to take many quizzes at the same time. I don't want to study anymore and take online classes, because as we are nursing students our knowledge and experience are important, and in the future, we will deal with patients and humans it's critical and from our responsibility. We did not get anything from online teaching it's different when we were in the college*.

Another student echoed the following:

*There was no understanding for the situation we are facing as a students from the teaching staff no use from this distance learning because we aren't learning anything or understanding procedures and lectures and according to the exams we had continuously issue while we are doing the exams in blackboard it will suddenly close for us even if we entered the quiz early and the teacher will not allow us to resit for the quiz even if it was a technical problem, what happened for us this semester was not fear at all*.

*There are problems such as the spacing that prevents us from studying with friends and reviewing lessons in the library*….

### 3.3. Challenges of Online Learning

Respondents cited several challenges related to online learning. These included Internet connectivity, teacher-related issues, strategy, and student issues. Some also reported that the home environment was not conducive to online learning. Some of the respondents had this to say.

#### 3.3.1. Connectivity Issues

*We faced many problems in the Internet breakdown and what prevented us from entering the lectures are those electronic problems that occur in computers and phones, we now miss chairs, blackboards, the doctor, the way of the university, and our lively minds*.

#### 3.3.2. Teacher-Related Issues

*Misunderstanding teachers feel the student have enough time each exam followed by other. We can't practice and work with our hands*.

*If the professor not well prepared, there will be a big mess*.

#### 3.3.3. Student Issues

*I did not have a chance to practice the skills, and some of the skills I am really struggling with and I did not fully get it (because I am a visual person) I need to see in order to understand so it was so difficult for me to understand the procedures*.

*Using a computer all the time while I'm used to using books*.

*Disturbances from family and siblings during online classes*.

### 3.4. Possible Solutions

Respondents cited a number of possible solutions or strategies to enhance online clinical instruction. These were system-related, teacher-related, and student-related solutions. The following are narrations from some of the respondents.

#### 3.4.1. System-Related Solutions

*Technical support …. Giving lectures suitable for this divergence, because it is difficult for us to understand the information via an electronic screen …. Rather, we should go to our university and study our lectures*.

*Developing the study with different and advanced capabilities, and changing the blackboard program to a college-specific program only to make the pressure on it lighter*.

*Obliging the student to purchase the equipment she needs for the laboratory for training and applying what she has studied at home…*.

#### 3.4.2. Teacher- and Student-Related Solutions

*In my opinion, I think lab course should be taken in lab classes, students should practice and apply the work directly to get better experience and become more professional*.

*Decrease the hours of studying and do other activities maybe researches depending of the same learning and knowledge and we do presentation discussing about it*.

*I think the explanation method must be developed to be practical in front of the student in order for the information to be well established in the student's mind*.

#### 3.4.3. Student-Related Solutions

*Provide chance to communicate students with each other through university website and creating work groups*.

*I think students should form study groups to study together and give each other support*.

## 4. Discussion

### 4.1. Demographic Data

This study was conducted with 25 undergraduate students. All participants were female because the campus is an all-female tertiary institution. All participants were Native Emiratis. The majority of the students were in the second year of the Bachelor of Nursing program. These are the students who have their first experience with clinical hospital-based learning. Inevitably, this cohort was bound to face a host of challenges as they tried to grasp clinical concepts as well as adjusting to the new concept of online clinical education. Traditionally, clinical teaching at the college is either delivered in the simulation laboratories or in healthcare institutions during clinical placement.

### 4.2. The Unfamiliar Experience

The sudden transition of teaching and learning modes had negative effects on work-life balance and mental health status and created challenges in copying with online teaching. The problems were compounded by the absence of well-developed infrastructure for the online classes, coupled with the extended and unprecedented period of the pandemic. Most of the respondents cited that the experience of online clinical learning was unfamiliar. Similarly, students in other studies have also echoed the same sentiments [16–18]. In addition, students expressed that they missed out on clinically based experiences. Though simulations and online clinical teaching may help achieve some objectives, they still fall short of the ability to allow students to access the complex situationism of workplace learning experiences [19]. Nursing education focuses on the utilisation of cognitive, affective, and psychomotor learning domains through structured healthcare education [20]. Unfortunately, this might not be adequately covered solely by online interactions. However, the traditional teaching methods were not suitable amid the COVID-19 pandemic [21]. Besides the learners, online clinical teaching can also be unfamiliar to faculty [22]. Nevertheless, practical learning with direct guidance and instruction from lecturers as well as with real patients stands to be the best approach and key in clinical education. This also boosts students' confidence [23].

Arguably, asynchronous online learning activities bring the convenience of fitting around students' lifestyles. However, this approach reduces the interaction between the teacher and the student and, consequently, significantly compromises student engagement and satisfaction [24]. Some respondents in our study also cited that they preferred to have more interaction with their tutors to enhance their understanding. Instructors must make all efforts to be present for the students online. There should be promotion, among students, of collaborative learning methods and dialogue to facilitate understanding, development of skills, and construction and application of knowledge [24].

### 4.3. Challenges of Online Learning

Participants in our study quoted several challenges with online learning. These were classified into the connectivity, teacher, strategy, and student issues. Regarding connectivity, respondents bemoaned slow and erratic connections that disrupted the flow of online sessions. A study conducted in Pakistan reported similar findings. Faculty delivering online medical education bemoaned the lack of adequate training and institutional support, erratic Internet connections, and poor student participation [25]. There was also a lack of understanding of the unique dynamics of online education, and this compromised the quality of teaching [25]. Similarly, another study conducted in Nepal among nursing students reported almost two-thirds (63.3%) of students having connectivity problems and another 63.2% having electricity problems [26]. Almost half of the faculty (42.3% and 48.1%) had electricity and Internet problems, respectively [26]. Currently, the college mainly uses the BlackBoardR as the main platform for teacher student interactions, with an option for Microsoft TeamsR. Other platforms, such as WhatsAppR, ZoomR, FacebookR, and PadletR, have also been used [9]. However, there must be reliable ways of transmitting information, particularly with adequate bandwidth and access to suitable devices for learners and educators alike. Ideally, student resources for learning should be freely available [9].

Respondents in our study reported that teachers gave inadequate explanations while they overloaded students with information. They complained that exams and other assessments were not well communicated to them and were difficult. The changes from conventional teaching to online teaching require good preparation for faculty to adapt to the paradigm shift [27]. Though the communication skills are similar to those for classroom teaching, teachers are not only required to be content experts but also pedagogical and technological personnel. With no prior training for the staff, delivery and content may be significantly compromised [28]. Online teachers usually face challenges in an effort to engage students on a virtual platform while managing instructional time and space as well as mastering virtual management techniques.

In addition to the connectivity issues, respondents complained about lack of hands-on experience and too much reliance on videos. Clinical sessions were reported to be generally long, and some students found it challenging to spend the long hours glued to the computer for the online sessions. There is also a potential of creating a “digital divide” between the students who can afford and those who cannot afford efficient Internet services [24]. Those who cannot afford are likely to be compromised as they tend to face disruptions during classes. Technology, as good as it is, should enhance, rather than detract, learning, teaching, and assessment of students [24].

Other participants cited isolation from friends during the study. Studies conducted earlier yielded similar results [29, 30]. The authors concur that online instruction can be a lonely experience, and social presence and interaction can potentially combat this challenge [29, 30]. It is argued that friendships, personal identity development, exposure to diversity, and self-care skills are important aspects of university life [31]. The development of these friendships is impeded by the sole online environment [31]. The problem, according to the respondents in our study, was compounded by erratic connections and certain gadgets that were difficult to use for online sessions. Without much interaction with peers and the teachers, respondents in our study consequently reported that it was difficult to self-evaluate regarding clinical competences.

Though asynchronised online learning experiences bring the liberty of working in the home and fitting in family commitments [24], some respondents in our study cited a lot of disturbances from family during online sessions. Several studies have also confirmed the negative influence of the home environment on learning [32–35]. Nursing students in a study conducted at a university in Texas, United States, also reported lack of motivation and too many family distractions in the home environment [32]. It is crucial to make the home environment conducive to learning. Some scholars suggest a personalised environment with very little distractions which addresses all the students' needs, good lighting, and reasonable comfort for the student at home [36].

### 4.4. Possible Solutions

Several suggestions to mitigate the challenges of online clinical learning were given by the respondents. These were subdivided into system-related, teacher-related, and student-related solutions. However, it should be emphasised that challenges of online learning require a multidisciplinary and multisectoral approach, with liaison among universities, schools, postgraduate training bodies, regulators, and employers [9].

Respondents in our study suggested several solutions that the system can implement. These included reverting to campus learning, modification of exams, development of college specific programs, and allowing students to purchase their own equipment for clinical teaching. The college has since reverted to campus-based and hospital-based clinical teaching. This was done with great caution and strict adherence to COVID-19 regulations to prevent the spread of the infection and promote the safety of both faculty and students. Some institutions have successfully redesigned and delivered face-to-face objective structured clinical examinations (OSCE) with strict adherence to COVID-19 regulations [37]. Some have used borderline regression methods to set standards for small cohorts [38, 39]. However, this requires resources and expertise and might not be feasible in resource-limited settings. Some universities, such as ours, with multiple campuses, have developed college-wide guidelines for virtual simulation as a substitution for a traditional face-to-face clinical session [32]. The process requires extensive consultation and identification of regulatory requirements and limitations for clinical experiences to develop evidence-based recommendations for simulation [32].

Teleteaching technologies have become very important in clinical education, particularly during the pandemic era [5]. Some institutions have explored the use of technology-enhanced assessment solutions for online exams by delivering “online OSCEs,” with structured oral examination components [9]. Some have successfully conducted OSCEs on ZoomR [40]. A study conducted in the UAE discovered that an online platform, Microsoft TeamsR, is feasible and effective in remote simulation-based communication skills training [41]. The college under study also uses Microsoft TeamsR, and this can be used to experiment other skill-training programmes. There is a need for modification of nursing curricula to include the heightened use of high-fidelity simulation to aid timely completion required for clinical rotations [32, 42]. The utilisation of virtual platforms can potentially support students' shift from being passive recipients of information to active participants in their own learning [24].

In addition, teachers need to receive further training in the use of virtual platforms to enhance student understanding of important concepts [4, 23, 43]. They need to partner with video game developers, the military, and others who are working at the forefront of augmented and virtual reality [4]. Some institutions have adopted livestreamed ward rounds for clinical teaching of medical students [44]. It must be stressed, though, that not all material for clinical learning can be delivered virtually [4].

Some respondents suggested the formation of student workgroups and generally reading more widely. A recent narrative review has also emphasised self-directed learning on the part of the student [45]. Webinars have also become popular as they involve larger groups of participants at a time [46]. Group discussions on multiple social media platforms are also being conducted by multiple educational bodies. The importance of moral support and collegiality in higher education cannot be overemphasised. Arguably, students need to read more widely and embrace new teaching strategies that have been cited as enhancements to virtual learning [43, 47]. These include use of case studies, activities that require critical thinking, problem-basedactivities, and active student engagement [48]. In support of this, Dedeilia et al. have emphasised the importance of small group problem-based learning (PBL), case presentation, and discussion in online education to engage students amid [47].

## 5. Conclusion

Students in our study had different perceptions on online clinical learning. Major themes identified were the unfamiliarity of the experience, the challenges faced by learners, and the possible solutions. In terms of education, several recommendations are hereby proposed. Nursing institutions, ours included, must develop flexible education systems that will be able to withstand and/or quickly recover from crisis and other unforeseeable circumstances [49]. Healthcare profession educators need to share learning resources across the global community as well as keep records of responses and innovations for future reference because they may shape future education and training [9, 23, 31].

Regarding practice, healthcare training institutions not only have the mandate to train professionals but are also central agents in providing the much-needed workforce for entire nations [9, 50]. As such, this mandates the institutions to graduate consistent numbers of professionals each year. Some have suggested graduating health professionals early or to time and then subsequently providing intensive initial workplace supervision and mentorship [9]. Addressing the disruptions imposed by the COVID-19 pandemic will require a multisystem and coordinated approach. Moreover, hospitals must strengthen internship programmes for newly graduated nurses to ensure that they catch up with all required skills and information.

Since the institution reverted to face-to-face instruction, there is no empirical evidence to suggest any poor performance of students in exams owing to the disruptions imposed by the pandemic. Several measures were taken to ensure quality learning, and these included increased clinical hours for students on clinical placements and increased utilisation of simulation laboratories to improve skills. A list of essential skills was developed by the college, and students were taught in both clinical settings and simulation laboratories to enhance their understanding. Therefore, the recommendation regarding research is that studies examining the difference in student performance before, during, and after the COVID-19 pandemic should be conducted.

Though our study gives important insight into students' perceptions on online clinical instruction, the study has its limitations. Data were collected online, and we could have missed nonverbal cues that can add meaning to participant responses. The semistructured questions used for data collection could have limited the responses of participants. However, the importance of being truthful on the part of the participants was stressed in the consent document. To mitigate researcher bias, because all the researchers are instructors in the same institution, reflexivity was observed throughout the research process. Member checking, prolonged engagement, and thick description of events were also utilized to minimise researcher bias.

## Figures and Tables

**Table 1:** Demographic data (*n* = 25).

Variables	Frequency	Percentage
Gender		
Female	25	100
Campus		
Ajman	25	100
Age		
17–24	22	88.0
25–32	1	4.0
33–40	2	8.0
Level of study		
1	1	4.0
2	11	45.0
3	8	32.0
4	5	19.0

**Table 2:** Themes, subthemes, and codes.

Main themes	Subthemes	Codes
The unfamiliar experience	Unfamiliar experience	Unfamiliar teaching methods
		Nonstimulating teaching environment
		Social isolation
		Lack of moral support
		Lack of mental preparation for online learning amid a global pandemic

Challenges of online learning	Connectivity issues	Numerous class interruptions due to network issues
		Lagging connections
	Teacher issues	Inadequate explanations of concepts
		Lack of fairness in student evaluation
		Poor communication regarding exams and other assessments
		Information overload on students
		Difficult exams
	Strategy issues	Lack of hands-on experiences
		Too much reliance on videos and pictures that are not adequate to enhance understanding of clinical concepts
		Inefficiency of the blackboard interface
		Frustrations of using the computer all the time for learning purposes
		Incompatibility of some electronic devices with online learning
	Student issues	Lack of interaction with peers
		Difficulty understanding explanations of practical concepts
		Hard to self-evaluate regarding clinical competences
	The home environment	Too many distractions in the home

Possible solutions	System-related solutions	Reversion to on-campus learning
		Modification of exams to suit online teaching and learning
		Development of college specific programs
		Allowing students to purchase equipment for clinical learning
	Teacher-related solutions	Giving more clear explanations
		Introduction of more case scenarios (case-based learning)
		Upholding fairness in student evaluation
	Student-related solutions	Formation of student workgroups and study groups
		Reading more widely

## Data Availability

The data used to support the findings of this study will be available from the corresponding author upon request.
